# Differential effect of whole-ear shading after heading on the physiology, biochemistry and yield index of stay-green and non-stay-green wheat genotypes

**DOI:** 10.1371/journal.pone.0171589

**Published:** 2017-02-03

**Authors:** Qing Li, Shengfu Zhong, Sifan Sun, Syeda Akash Fatima, Min Zhang, Wanquan Chen, Qianglan Huang, Shengwen Tang, Peigao Luo

**Affiliations:** 1 Provincial Key Laboratory of Plant Breeding and Genetics, Sichuan Agricultural University, Chengdu, Sichuan, China; 2 Department of Biology and Chemistry, Chongqing Industry and Trade Polytechnic, Fuling District of Chongqing, China; 3 State Key Laboratory for the Biology of Plant Diseases and Insect Pests, Institute of Plant Protection, Chinese Academy of Agricultural Sciences, Beijing, China; Institute of Genetics and Developmental Biology Chinese Academy of Sciences, CHINA

## Abstract

Two winter wheat cultivars (the functional stay-green CN12 and non-stay-green CN19) were used to investigate the effects of ear-shading on grain yield and to elucidate the differential mechanisms of different cultivars. The photosynthetic parameters, chlorophyll fluorescence, antioxidant enzyme activities, and chlorophyll contents were measured 0, 15 and 30 days after heading (DAH) under both shaded and non-shaded conditions. The final grain-yield index was also measured. Shading had a smaller effect on the net photosynthetic rate (*Pn*), intercellular CO_2_ concentration (*Ci*), stomatal conductance (*Gs*), maximal photochemical efficiency of PSII (*Fv*/*Fm*) and coefficient of non-photochemical fluorescence quenching (*qN*) but a greater effect on both superoxide dismutase (SOD) and catalase (CAT) activities in CN12 than it did in CN19. Shading slightly altered the timeframe of leaf senescence in CN12 and may have accelerated leaf senescence in CN19. Moreover, shading had only a small effect on the weight of grains per spike (WGS) in CN12 compared with CN19, mainly resulting from the number of grains per spike (NGS) rather than the 1000-grain weight (SGW). In conclusion, the flag leaves of functional stay-green wheat could serve as potential “buffers” and/or “compensators” for ear photosynthesis, which is actively regulated by the antioxidant enzyme system and prevents yield loss. Thus, a functional stay-green genotype could be more tolerant to environmental stress than a non-stay-green genotype.

## Introduction

Some authors have proposed that crop production needs to double by 2050 to meet the food demands of the rapidly increasing population, and numerous authors have suggested that the most sustainable path for achieving food security is increasing crop yields rather than clearing more land for crop production [1]. Therefore, crop-yield improvement is the most important aim of global long-term cultivation efforts. Wheat is the most widely grown crop in the world, providing more than 20% of the caloric intake for 4.5 billion people, and is the second most widely grown crop in China, after rice [2]. The average wheat yield has significantly increased, with total production reaching 126 million tons in 2014, mainly due to many research efforts aimed at cultivating new wheat varieties [3].

However, achieving further increases in wheat yield is difficult using traditional genetic methods but is possible by improving the functions of and links among various organs throughout the entire plant. Different plant organs exhibit diverse tasks and biological functions. For example, one of the crucial functions of leaves as a “source” is to synthesize energy-rich molecules, whereas the developing seeds can generally be defined as a carbohydrate “sink” [4]. The source/sink relationship is a dynamic balance [5], and a better understanding of this relationship using different wheat genotypes would contribute to both the elucidation of yield formation mechanisms and systematic yield improvement. Some authors have suggested that wheat genotypes that differ in both the onset and the speed of leaf senescence are ideal for analyzing the source/sink relationship [6–8].

In addition, some organs, such as the wheat plant’s ear, cannot be simply defined as a source or sink. The wheat ear is generally treated as a sink, especially during the grain-filling stage, when the developing seeds are growing; however, many parts of the ear, including the green awn, husk and axis, are photosynthetically important [9]. Ear photosynthesis contributes significantly to grain yield under either water deficit or irrigation and therefore represents a “buffer” for maintaining grain yield, especially under “source” limitation, the most frequent condition experienced by modern cultivars [9].

Grain yield is strongly related to light intensity; therefore, some authors have assumed that shading reduces wheat yields by reducing both the leaf area index and photosynthesis rate in wheat leaves [10,11]. In contrast, other authors believe that shading may affect the crop canopy, increase the leaf area index, delay leaf senescence and increase photosynthesis rates, increasing wheat grain yield [12,13]. Nevertheless, whether the yield increases or decreases depends on shading and weather conditions [14,15], although important varietal characters have been ignored.

Both high light conditions and low light conditions due to shading generally lead to premature leaf senescence and cause great yield losses in winter wheat [12,16]. Studies have revealed natural genetic variation among genotypes with regard to the degree and rate of plant response to light intensity [17]. However, wheat leaf senescence shortens the efficient photosynthetic period and severely decreases yields [6]. These findings indicate that genetic variations related to light susceptibility that induce leaf senescence among different varieties exist. Moreover, delaying leaf senescence could be an effective strategy for increasing cereal production in the future [18].

Stay-green mutants, which typically exhibit delayed leaf senescence, can sustain photosynthetic competence for a longer period and maintain the supply of assimilates to the developing grains [19]. Various authors have reported stay-green mutants of durum and wheat [20,21]. However, the first reported stay-green wheat cultivars were CN12, CN17 and CN18 [22], which are widely grown in many regions (especially in southwest China) due to their high yield [5,6] and resistance to diseases such as stripe rust [23]. All of these cultivars arose from wheat-rye 1BL/1RS translocations [24], and they exhibit a similar functional stay-green phenotype. These cultivars were released into southwest China in 2003 by a group led by Professor Zhenglong Ren [5,6,25]. Functional stay-green wheat cultivars might be useful plant material for elucidating the dynamic relationship between sources and sinks because these cultivars are generally sink-limited cultivars compared with numerous other modern wheat cultivars [6,9]. Unfortunately, differences in response to shading between the two types of wheat varieties remain unknown.

Therefore, we used the functional stay-green sink-limited cultivar CN12 and the source-limited modern wheat cultivar CN19 in the present study. After heading, ear-shading was performed, and no shading was used as the control. Physiological and biochemical indices, chlorophyll components and contents, chlorophyll fluorescence and grain yields were measured. The main objectives were to investigate the overall variation due to fluctuations of these parameters, to compare the differences in the tendencies of these parameters to change between the stay-green and non-stay-green genotypes and to determine the source/sink relationship in different genotypes.

## Materials and methods

### Growth of plant materials and shading treatments

Two wheat cultivars (stay-green CN12 and non-stay-green CN19) were used in this study. CN12 is a functional stay-green phenotype that can delay leaf senescence and maintain photosynthetic competence for a longer period during the grain-filling stage [22,25]. CN19 is widely grown in the southwestern region of China due to its high yield and disease resistance [26,27] and has a similar maturity date as that of CN12 [28].

These two cultivars were sown on November 1, 2015, in a field at the Wenjiang Agricultural Research Station of Sichuan Agricultural University in southwest China. A randomized complete block design with three replications was used. The plots consisted of 2-m-long rows with 0.3-m spacing between rows and 10-cm spacing between plants within rows. In each replication, the spikes of 12 plants of each genotype were covered using opaque ventilated bags at the heading stage from March 13 to 15, 2016, while an additional 12 plants were used as the non-shaded group for comparison. Plants with similar growth and developmental progress were randomly chosen from plots. Three of the 12 plants were used to measure photosynthetic parameters and chlorophyll fluorescence 0, 15 and 30 days after heading (DAH) and to subsequently measure the yield index. The other plants were employed for harvesting flag leaves for measurements of biochemical parameters at various time points.

### Antioxidant enzyme activity assays

From each sample, 0.2 g of fresh leaves was homogenized in 1.6 mL of extraction buffer containing 50 mM potassium phosphate buffer (pH 7.8) and 1% polyvinylpyrrolidone. After the homogenate was centrifuged at 10,000 g for 20 min at 4°C, the supernatant was immediately used to measure enzyme activities.

Superoxide dismutase (SOD) activity was measured using a previously described method that quantifies the ability to inhibit the photochemical reduction of nitro blue tetrazolium chloride (NBT) [29]. The reaction solution included 75 μM NBT, 50 mM phosphate buffer (pH 7.8), 2 μM riboflavin, 0.15 mM EDTA-Na_2_, 13 mM methionine and 10 μL of enzyme extract. The transparent test tubes containing the reaction mixture were illuminated with light irradiance of 4000 lx for 20 min. A tube containing the same reaction mixture was placed in the dark as the blank, and a tube containing the solution without the enzyme incubated in the light served as the control. The absorbance of the solution was determined at 560 nm. One unit of SOD activity was defined as the amount of enzyme that inhibited the reduction of NBT in the light by 50%. Peroxidase (POD) activity was measured with guaiacol and H_2_O_2_ at 470 nm after guaiacol oxidation [30]. The assay mixture contained 50 mM phosphate buffer (pH 6.1), 0.4% H_2_O_2_, 1% guaiacol and 10 μL of enzyme extract in a final 3.0-mL solution. POD activity was expressed as the increase of the absorbance at 470 nm. Catalase (CAT) activity was determined based on the principle that changes in the absorbance of the reaction mixture are proportional to the breakdown of H_2_O_2_ [31]. The reaction mixture consisted of 0.15 M phosphate buffer (pH 7.8), 0.1 M H_2_O_2_ and 20 μL of enzyme extract in a total volume of 3.0 mL. After mixing and shaking, the absorbance of the reaction solution was immediately monitored for 2 min at 240 nm.

### Measurement of chlorophyll and malondialdehyde contents

The chlorophyll contents were determined via the acetone method described by Lichtenthaler [32]. A 0.2-g sample of fresh leaves was ground under 5 mL of ice-cold 80% acetone to extract chlorophyll, and the absorbance of the homogenate was then measured at 663, 645 and 470 nm. The malondialdehyde (MDA) contents were measured using the thiobarbituric acid (TBA)-based colorimetric method [33]. From each sample, 0.2 g of fresh leaves was ground with 1.6 mL of 10% trichloroacetic acid. The homogenate was then transferred to a tube and centrifuged at 10,000 g for 15 min. The reaction mixture containing 1.5 mL of the supernatant and 1.5 mL of 0.67% TBA was incubated at 100°C for 30 min. After cooling the mixture on ice, the absorbance of the supernatant was measured at 600, 532 and 450 nm.

### Measurement of photosynthetic indices

A portable photosynthetic system (Li-6400-02B, Li-Cor, Lincoln, NE, USA) with a red-blue light source was used to measure related physiological parameters in each plant. The conditions under which the measurements were performed were as follows: air temperature of 21 to 24°C; vapor pressure deficit (VPD) of 0.55 to 0.65 kPa; and actinic light intensity of 1000 μmol·m^-2^·s^-1^. Each measurement was performed in the center of the flag leaf and took approximately 1 min. The average value from three flag leaves was used as the value for the plant at that time point, and three plants were measured in each block. The values of the photosynthetic rate (*Pn*), stomatal conductance (*Gs*), intercellular CO_2_ concentration (*Ci*) and transpiration rate (*Tr*) were recorded.

### Measurements of chlorophyll fluorescence and the quantum yield of PSII

Chlorophyll fluorescence was measured as previously described [34]. The coefficient of photochemical chlorophyll fluorescence quenching (*qP*), the coefficient of non-photochemical fluorescence quenching (*qN)*, the maximum fluorescence in the light (*Fm’*), the variable chlorophyll fluorescence yield in the light (*Fv’*) and the actual photochemical efficiency of PSII (*Ф*_*PSⅡ*_) were directly measured using a modulated photosynthetic system (6400XT, Li-Cor, Lincoln, NE, USA) with a leaf chamber fluorometer (Li-6400-40; Li-Cor, Lincoln, NE, USA) under the following conditions: air temperature of 21 to 24°C; VPD of 0.55 to 0.65 kPa; and actinic light intensity of 1000 μmol·m^-2^·s^-1^. Because the maximum fluorescence (*Fm*) and variable chlorophyll fluorescence yield (*Fv*) must be measured under dark adaptation, the leaf samples were acclimated to the dark for 30 min to ensure the maximal photochemical efficiency of PSII (*Fv/Fm*).

### Investigation of grain yields

After manual harvesting, the seeds were oven dried, and the number of grains per spike (NGS), 100-grain weight (SGW) and weight of grains per spike (WGS) were determined.

### Statistical analysis

Significant differences in the mean physiological parameters, photosynthetic parameters and yield traits between the two genotypes (CN12 and CN19), two treatments (shading and non-shading) and three time points were determined via independent sample ANOVA tests, t-tests or multiple comparisons with IBM Statistical Package for Social Science (SPSS) 19 software (SPSS Inc., Chicago, IL), depending on the experimental design.

## Results

### Changes in photosynthetic parameters

**Fig 1:**
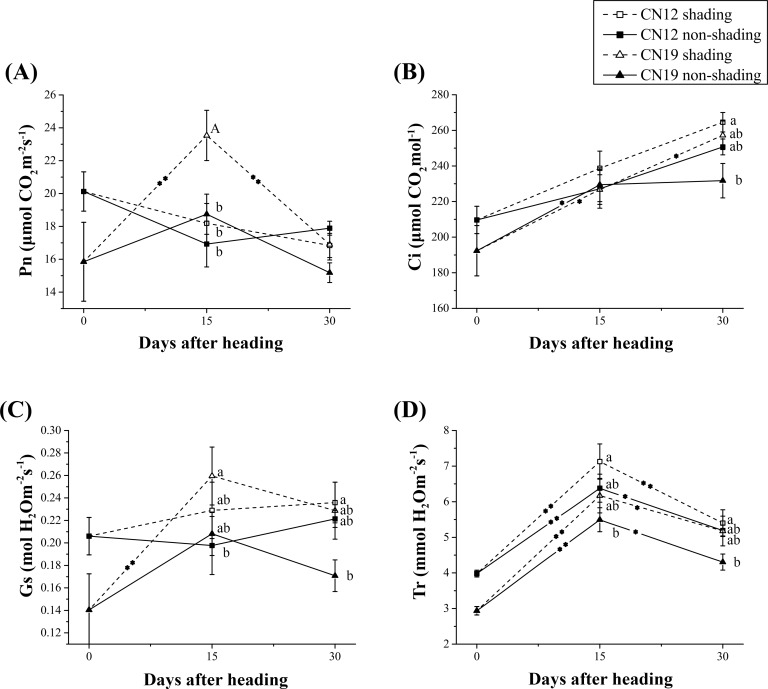
Changes in Photosynthetic Parameters Between the Stay-Green CN12 and Non-Stay-Green CN19 Wheat Cultivars Under Shading. (A), Net photosynthetic rate (*Pn*); (B), stomatal conductance (*Gs*); (C), intercellular CO_2_ concentration (*Ci*); and (D), transpiration rate (*Tr*). The bars represent the mean ± standard error (SE). Asterisks represent statistically significant differences, as follows: **P≤0.01 and *P≤0.05. Letters represent the probability of multiple comparisons of the means of different genotypes and treatments at each time point, as follows: capital letter, P≤0.01; lowercase letter, P≤0.05. An asterisk in the trend line represents the difference between two adjacent time points for the same genotype and treatment.

At the heading stage, significant differences in *Pn* were found between non-shaded CN12 and CN19, and the changes in *Pn* in non-shaded CN12 differed markedly from the changes in non-shaded CN19 after heading (Fig 1). In the non-shaded group, the *Pn* of CN12 markedly decreased from 0 DAH to 15 DAH and slightly increased from 15 to 30 DAH. In contrast, *Pn* markedly increased from 0 to 15 DAH and markedly decreased from 15 to 30 DAH (Fig 1A). In the shaded group, there was a minimal decreasing tendency of *Pn* in CN12, whereas the *Pn* of CN19 significantly (P≤0.01) increased from 0 to 15 DAH and then significantly (P≤0.01) decreased from 15 to 30 DAH (Fig 1A). In general, the *Ci* of both CN12 and CN19 increased after heading, and the change in *Ci* was significant in both non-shaded and shaded CN19 from 0 to 15 DAH (P≤0.05) and 15 to 30 DAH (P≤0.05), respectively (Fig 1B). The net changes in *Gs* were significant in shaded CN19 from 0 to 15 DAH (P≤0.01) (Fig 1C). All treatments showed similar sharp changes in *Tr* after heading (Fig 1D).

### Chlorophyll fluorescence parameters

**Fig 2:**
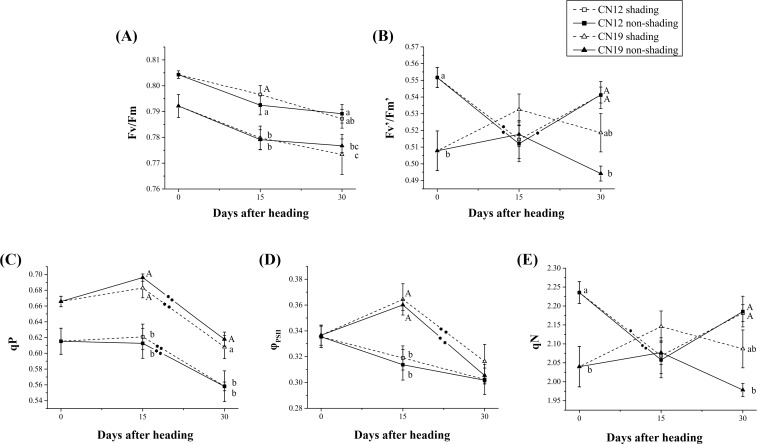
Changes in Chlorophyll Florescence Parameters Between CN12 and CN19 Under Shading. (A), Maximal photochemical efficiency of PSІІ in dark-adapted leaves (*Fv* /*Fm*); (B), efficiency of excitation capture by open PSII reaction centers (*Fv’/Fm’*); (C), photochemical quenching coefficient (*qP*); (D), quantum yield of photochemical energy conversion in PSII (*Ф*_PSII_); and (E), regulated non-photochemical energy loss in PSII (*qN*). Bars represent the mean ± (SE). Asterisks represent statistically significant differences, as follows: **P≤0.01 and *P≤0.05. Letters represent the probability of multiple comparisons of the means of different genotypes and treatments at each time point, as follows: capital letter, P≤0.01; lowercase letter, P≤0.05. An asterisk in the trend line represents the difference between two adjacent time points for the same genotype and treatment.

Although CN12 presented a significantly higher *Fv*/*Fm* (P≤0.05) than did CN19 at each time point after heading, the change tendency of *Fv*/*Fm* after heading was similar in the two genotypes. However, shading had a slight effect on the changes in *Fv*/*Fm* (Fig 2A). There was a significant difference in efficiency of excitation capture by open PSII reaction centers (*Fv’/Fm’*) at 0 DAH and in the total change tendency between CN12 and CN19. *Fv’/Fm’* decreased significantly (P≤0.05) from 0 to 15 DAH and then increased significantly (P≤0.05) from 15 to 30 DAH in non-shaded CN12, while no significant change in *Fv’/Fm’* was observed after heading in shaded CN19. In addition, shading had minor effects on *Fv’/Fm’* in both genotypes, with a slightly larger effect on CN19 than on CN12 (Fig 2B). All four treatments resulted in similar changes in *qP*, with a significant (P≤0.01) decrease observed in both the shaded and non-shaded genotypes (Fig 2C). However, the change tendency of *Ф*_*PSⅡ*_ in non-shaded CN12 was different from that in non-shaded CN19, and the change tendency of *Ф*_*PSⅡ*_ in the shaded group was similar to that in the corresponding non-shaded genotypes (Fig 2D). The net change in *qN* after heading was similar to that of *Fv’/Fm’*, and shading had a greater effect on *qN* in CN19 than in CN12 (Fig 2E).

### Chlorophyll components and contents

**Fig 3:**
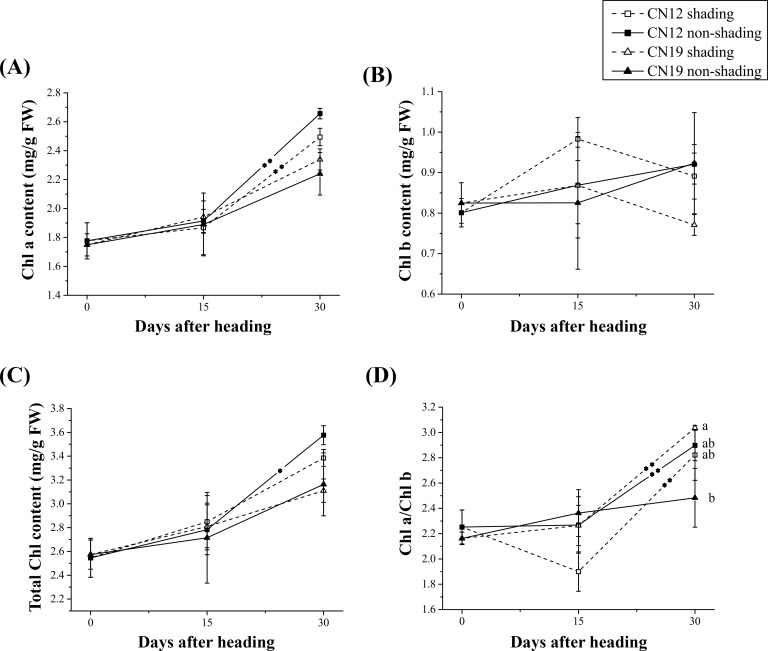
Changes in Chlorophyll Content Between CN12 and CN19 Under Shading. (A), Chlorophyll a (*Chl a*) content; (B), chlorophyll b (*Chl b*) content; (C), total chlorophyll content; and (D), *Chl a*/*b*. Bars represent the mean ± (SE). Asterisks represent statistically significant differences, as follows: **P≤0.01 and *P≤0.05. Letters represent the probability of multiple comparisons of the means of different genotypes and treatments at each time point, as follows: capital letter, P≤0.01; lowercase letter, P≤0.05. An asterisk in the trend line represents the difference between two adjacent time points for the same genotype and treatment.

The chlorophyll contents were measured in the flag leaves of CN12 and CN19 under both shaded and non-shaded conditions (Fig 3). The changes in both the chlorophyll a (*Chl a*) and total chlorophyll (*Chl*) contents generally exhibited similar change tendencies, with the *Chl a* and *Chl* contents markedly increasing from 15 to 30 DAH. The increases in the *Chl a* and *Chl* contents were sharper in CN12 than in CN19 under both shaded and non-shaded conditions (Fig 3A and 3C). In the non-shaded group, the two genotypes displayed a similar effect of shading on the chlorophyll b (*Chl b*) contents as well as a similar change tendency of *Chl b* (Fig 3B). Further analysis showed that the effect of shading on *Chl b* in CN12 was greater than that in CN19, and the variation in the *Chl a*/*Chl b* ratio also differed between the two genotypes under shaded conditions (Fig 3D).

### Changes in biochemical parameters

**Fig 4:**
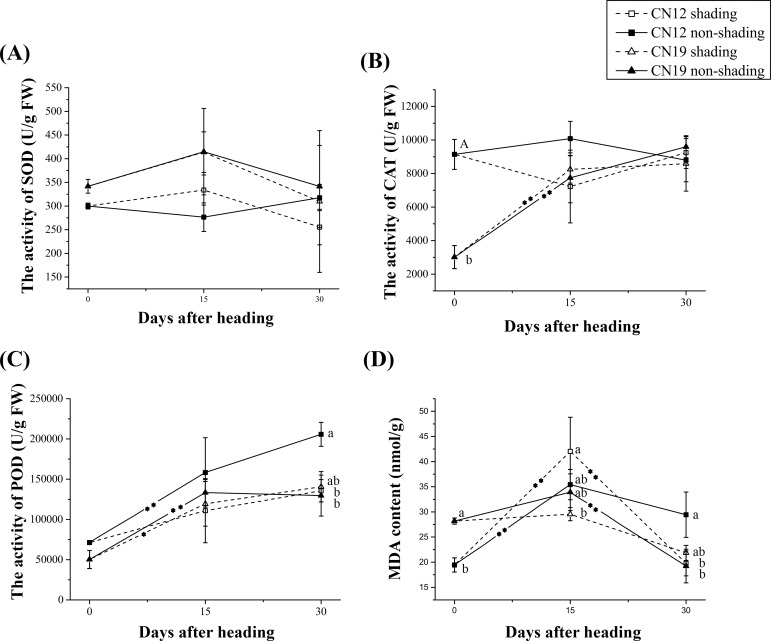
Changes in Antioxidant Enzyme Activity and Related Biochemical Parameters in CN12 and CN19 Under Shading. (A), superoxide dismutase (SOD); (B), catalase (CAT); (C), peroxidase (POD); and (D), malondialdehyde (MDA). The bars represent the mean ± (SE). Asterisks represent statistically significant differences, as follows: **P≤0.01 and *P≤0.05. Letters represent the probability of multiple comparisons of the means of different genotypes and treatments at each time point, as follows: capital letter, P≤0.01; lowercase letter, P≤0.05. An asterisk in the trend line represents the difference between two adjacent time points for the same genotype and treatment.

The change in the SOD activity in non-shaded CN12 differed from that in non-shaded CN19 (Fig 4A). In CN12, the SOD activity decreased from 0 to 15 DAH and increased from 15 to 30 DAH. In contrast, in CN19, the SOD activity increased from 0 to 15 DAH and decreased from 15 to 30 DAH in CN19. In the shaded group, CN12 and CN19 presented a similar change tendency in SOD activity (Fig 4A). Shading had greater effects on both CAT and POD in CN12 compared with those in CN19 (Fig 4B and 4C). However, the change tendency of the MDA contents was similar in all four samples; similarly, shading had a greater effect on MDA in CN12 compared with that in CN19 (Fig 4D).

### Phenotypic observations and grain yields

CN12 exhibits a stay-green phenotype based on continuous multi-year records, similar to its sister line CN17 [6]. The control genotype CN19 displayed visible yellowing of the leaves at 30 DAH, while the leaves of CN17 retained normal green coloration at the same time. Soon thereafter, the CN19 plants began to undergo a normal senescence progress, with the leaves showing senescence before the stems. In the CN12 plants, the senescence of the leaves and stems began almost simultaneously at 43–44 DAH, showing slow progress. CN19 exhibited a higher grain index (including NGS, WGS and SGW) than did CN12 under both shaded and non-shaded conditions, with the exception of a lower SGW in shaded CN19 compared with shaded CN12 (Table 1).

**Table 1:** Effects of Shading Treatment on Grain Production and Weights in CN12 and CN19.

	Number of grains per spike			Weight of grains per spike (g)			1000-grain weight(g)		
Genotypes	Shaded	Non-Shaded	Effects	Shaded	Non-Shaded	Effects	Shaded	Non-Shaded	Effects
CN12	32.92±1.850	37.81±1.975	12.93%	1.29±0.085	1.55±0.092*	16.77%	39.09±1.392	40.92±1.213	4.47%
CN19	43.19±1.992^‡^	49.96±1.437*^‡^	13.55%	1.65±0.056^‡^	2.13±0.054** ^‡^	22.54%	38.55±1.496	42.62±0.594*	9.55%

The data are presented as the mean ± SE. The percentage values are the effects of the shading treatment on each index of each genotype.

*, ** indicate significant differences between the shaded treatment and the non-shaded treatment in the same genotype at P = 0.05 and P = 0.01, respectively.

^‡^ indicate significant differences between the two genotypes under the same treatment at P = 0.01.

## Discussion

Previous studies have shown that CN12 plants that are not subjected to environmental stress exhibit higher photosynthetic competence during the grain-filling stage than do MY11 plants, especially during the later stages of leaf senescence [25]. Further research demonstrated that CN12 and its sister line CN17 also present high resistance to paraquat-induced photooxidative stress [35]. However, the response of wheat stay-green genotypes to shading stress remained unknown. The leaves of CN12 plants maintained normal green coloration, while the leaves of CN19 plants began to yellow at 30 DAH, approximately 21–22 days after anthesis, when the senescence of wheat leaves began [7]. Ear-shading had a minor effect on the *Pn*, *Ci* and *Gs* of CN12 flag leaves but had a greater effect on these parameters in CN19 (Fig 1). *Gs* and intra-photosynthetic apparatus activity are two major factors that generally influence *Pn* [36]. A comprehensive comparison of the data presented in Fig 1 indicated that under ear-shading conditions, the predominant factor regulating *Pn* in the control CN19 plants might have been *Gs* rather than the intrinsic photosynthetic capacity. Further analyses of the differential effects of shading on the change tendency of *Pn* indicated that leaf senescence occurred more rapidly under shading than under non-shaded conditions, regardless of the possibility that the onset of leaf senescence was not accelerated (Fig 1A). In fact, the *Gs*-mediated plant response to environmental stress or pathogens is the most common passive mechanism associated with plant fitness [37,38]. Thus, the data suggested that the functional stay-green wheat cultivar CN12 responded effectively to ear-shading stress through the inherent photosynthetic apparatus and delayed leaf senescence under normal field conditions. Moreover, a comparison of the change propensity of *Pn* between shaded CN12 and non-shaded CN12 plants also indicated that shaded CN12 maintained a higher *Pn* level than did non-shaded CN12 in the later grain-filling stage (Fig 1A), which might have significantly contributed to the minor influence of shading on the yield indices, especially for SGW (Table 1). Therefore, we concluded that under ear-shading conditions, the stay-green genotype could delay the onset of leaf senescence and maintain higher photosynthesis with a slower rate of leaf senescence, in contrast to the non-shaded stay-green plants during leaf senescence.

Shading induces premature plant leaf senescence, especially under weak natural light conditions [39]. Complete shading (i.e., darkness) is an important factor contributing to leaf senescence [12]. In the present study, the whole ear was covered after heading using opaque ventilated paper bags, which influenced leaf senescence in view of photosynthetic parameters (Fig 1). However, the difference in the influence of ear-shading between the photochemical efficiency of PSII and the efficiency of excitation was captured by open PSII. Non-shaded CN12 exhibited a higher *Fv*/*Fm* and a higher *qP*, and the difference between CN12 and CN19 was significant (P≤0.05). In addition, ear-shading had only a minor effect on *Fv*/*Fm*, *qP* and *Ф*_*PSⅡ*_ in both genotypes (Fig 2A, 2C and 2E). In CN12, ear-shading had a slight effect on both *Fv’/Fm’* and *qN*, while a greater effect on these parameters was observed in CN19 (Fig 2B and 2D). The changes in chlorophyll fluorescence parameters in the stay-green CN12 indicated that leaf senescence was delayed until the later stage of grain-filling under shaded conditions, which contributed to the mechanism of closing a portion of the active center of PSII (e.g., decreasing the efficiency of excitation capture by open PSII) in the early stage. However, this mechanism was absent in non-stay-green CN19 under natural conditions. Moreover, the increase in the efficiency of excitation capture by open PSII in the early stage may have been responsible for the more rapid leaf senescence in shaded CN19.

Changes in chlorophyll components and contents play a major role in leaf senescence [40]. Although shading had a similar effect on chlorophyll components and contents in the present study, the effect in CN12 was more obvious than it was in CN19 (Fig 3). There was a marked difference in *Chl* b between shaded CN12 and shaded CN19 at 30 DAH (Fig 3B). Because *Chl* a and *Chl* b are important components of light-harvesting complex II (LHCII), the data suggested that the LHCII of PSII may have been retained in both shaded and non-shaded CN12, possibly due to state transitions in the membrane architecture [41] and the reconstruction of the photosynthetic apparatus, similar to what occurs in its sister line CN17 during the early stage of leaf senescence [7]. Without considering details and mechanisms, the stay-green genotype clearly exhibited a stronger ability to resist senescence caused by ear-shading than did the non-stay-green genotype.

SOD, POD and CAT are important components of a plant’s antioxidant system, and these indices, along with MDA, are common and useful parameters for evaluating a plant’s redox status [42]. High SOD, POD, and CAT activities accompanied by a low MDA content generally indicate a high antioxidant ability [43]. Ear-shading had greater effects on the SOD, POD, and CAT activities and MDA contents in CN12 than in CN19 (Fig 4). Non-shaded CN12 exhibited lower SOD activity and higher POD activity than did non-shaded CN19. Interestingly, significantly higher (P≤0.05) CAT activity and significantly lower (P≤0.05) MDA contents were found in CN12 than in CN19 under non-shaded conditions (Fig 4B and 4D). Shading not only had a greater effect on CAT activity but also altered the change tendency of CAT activity after heading in CN12. However, ear-shading had only a minor effect on CAT activity, and the change tendency of CAT activity was similar between shaded and non-shaded CN19 (Fig 4B). CAT is essential for the removal of H_2_O_2_ [6]. H_2_O_2_ is a versatile molecule that can act as a signal at non-toxic concentrations, damage plant cells, and accelerate senescence [44]. Therefore, the obvious decrease in CAT activity observed at 15 DAH in shaded CN12 (as shown in Fig 2B) might be explained by the adjustment of the H_2_O_2_ concentration to the ideal level as a signaling molecule. This decrease in CAT activity may have simultaneously caused slight membrane damage in shaded CN12. Thus, it can be concluded that in contrast to the non-stay-green genotype, the stay-green genotype exhibits an actively regulated mechanism for coping with reactive oxygen species (especially H_2_O_2_) under biotic or abiotic stress (where H_2_O_2_ might play its ideal role as a signaling molecule) without causing severe membrane damage.

The contribution of photosynthesis in the green parts of the ear can be substantial, as it may be responsible for 20–40% of the wheat grain yield [45]. In the present study, whole-ear shading caused a significant (P≤0.05) reduction of WGS in CN12 and a highly significant reduction in CN19 (P≤0.01) (Table 1). These results confirmed that wheat ear photosynthesis contributes greatly to grain yield with slight variation between different varieties, indicating that the flag leaves of the functional stay-green CN12 cultivar could serve as better “buffers” and/or “compensators” than those of the non-stay-green CN19 cultivar for the protection of the grain yield under whole-ear-shading conditions. In addition to exhibiting high disease resistance [23], CN12 displays a higher yield and has a significantly greater number of spikes per unit area than control plants [22]. CN12 also presents an obvious functional stay-green phenotype during leaf senescence with high photosynthetic competence in the later stage of leaf senescence [25]. These findings suggest that CN12 is a typical sink-limited cultivar. In contrast, CN19, which displays high resistance to diseases such as stripe rust [26,27], is a high-yielding cultivar mainly due to its larger ears and greater NGS and SGW compared with those of control plants [46]. The major yield loss observed under whole-ear shading after heading, especially in terms of total WGS, indicated that CN19 is a classical source-limited cultivar, which is the main type of modern wheat cultivar [9].

Furthermore, ear-shading affected yield components, including both NGS and SGW, with different intensities in CN12 and CN19 (Table 1). Finally, there was a smaller influence on the grain yield in the stay-green genotype (16.77%) than in the non-stay-green genotype (22.54%). Further analysis revealed that the minor yield loss observed in the stay-green genotype mainly resulted from the effect of ear-shading on NGS (12.9%) rather than on SGW (4.47%). The effects of ear-shading on both NGS and SGW were not significant in CN12, in contrast to CN19, which showed significant effects on both parameters (P≤0.05) (Table 1). Photosynthetic competence in the early grain-filling stage after heading is markedly positively correlated with sink strength [5,47]. Hence, the photosynthesis of juvenile ears in the early grain-filling stage after heading mainly determines the sink strength, weighted by NGS, which displays a source function. In contrast, in the later grain-filling stage, the fast-developing seeds in the spikes indicate that the ear acts completely as a sink. Therefore, the data in Table 1 indicate that the stay-green CN12 genotype exhibited higher photosynthetic competence in the later stage of leaf senescence (i.e., after 30 DAH).

In conclusion, the functional stay-green wheat genotype can delay leaf senescence during the grain-filling stage equally well under whole-ear-shading and non-shaded conditions. The flag leaves of the stay-green genotype might act as better “buffers” (inhibiting the reduction of grain yield) and “compensators” for ear photosynthesis such that the negative effect of ear-shading on grain yield is lower in the stay-green genotype than in the non-stay-green genotype. These processes are involved in the active regulation of photosynthetic competence by regulating the number of open PSII reaction centers, the changes in chlorophyll components and contents, the concentrations of reactive oxygen species through antioxidant enzyme activities (especially the H_2_O_2_ concentration regulated by CAT), and the signaling pathways in which H_2_O_2_ participates.
